# Effect of Germanium Substitution for Aluminium on the Properties of Calcium Fluoro-Alumino-Silicate Glass-Ceramics

**DOI:** 10.3390/ma19173570

**Published:** 2026-08-22

**Authors:** Kuopei Yu, Siqi Zhang, Yuhang Liu, Wen Ni

**Affiliations:** 1 School of Resource and Safety Engineering, University of Science and Technology Beijing, Beijing 100083, China; yukuopei@qingli.cn (K.Y.); hangliu2024@163.com (Y.L.); niwen@ces.ustb.edu.cn (W.N.); 2 Institute of Mineral Resources, University of Science and Technology Beijing, Beijing 100083, China

**Keywords:** germanium, calcium fluoro-alumino-silicate glass, fluorapatite, anorthite, glass-ceramics

## Abstract

This study systematically explores the impacts of germanium (Ge) substitution for aluminium (Al) on the structure and properties of calcium fluoro-alumino-silicate glass (4.5SiO_2_-3Al_2_O_3_-1.5P_2_O_5_-3CaO-2CaF_2_) and its derived glass-ceramics, aiming to mitigate Al-induced biological toxicity in the materials. Glass samples with 0–60 mol% Ge substitution were prepared via melting–quenching, and comprehensively characterized by X-ray diffraction (XRD), differential scanning calorimetry (DSC), and scanning electron microscopy–energy-dispersive X-ray spectroscopy (SEM-EDS). Results show that the base glass contains three crystalline phases: fluorapatite (FAp, Ca_5_(PO_4_)_3_F), anorthite (CaAl_2_Si_2_O_8_), and aluminium phosphate (AlPO_4_). Ge^4+^ integrates into the glass network as [GeO_4_] tetrahedra without forming independent Ge-based phases; with rising Ge substitution ratio, FAp mass fraction increases from 36% to 65%, anorthite decreases from 46% to 18%, and glass transition temperature (Tg) drops from 678 °C to 617 °C. High Ge substitution (≥40 mol%) triggers network relaxation and spherical particle formation, and local Ca/P ratio reduction drives FAp morphological reconstruction, while low Ge content (≤20 mol%) promotes regular phase crystallisation. The optimal Ge substitution level is 20 mol%, which cuts Al content by 20% to alleviate toxicity, moderately reduces enthalpy for improved sinterability, and realises synergistic mechanical reinforcement via FAp, anorthite and AlPO_4_. This material integrates low toxicity, favourable sinterability and excellent bioactivity, providing a novel strategy for developing low-Al, high-bioactivity glass-ceramics for dental and bone repair applications.

## 1. Introduction

Calcium fluoro-alumino-silicate glass (4.5SiO_2_-3Al_2_O_3_-1.5P_2_O_5_-3CaO-2CaF_2_) has been extensively utilised in dental restorations and bone cements due to its exceptional bioactivity [1,2]. A distinctive characteristic of this particular ionic glass is its capacity to undergo a process of crystallisation, resulting in the formation of glass-ceramics that contain apatite. This phenomenon is primarily attributed to the similarity between the apatite phase and the biological apatite found in teeth and bones [3,4]. Such bioactive glasses have been shown to form tight bonds with both soft and hard tissues [5,6]. During the process of implantation, a bioactive carbonated hydroxyapatite layer is formed on the glass surface, thereby serving as an interface for tissue attachment. The hydroxyapatite layer between the glass and tissues exhibits considerable mechanical strength, thereby enabling resistance to significant mechanical forces. The design and characterisation of such glasses play a crucial role in the development of novel biomedical materials, where the properties of glass-ceramics are strongly dependent on their composition, crystallisation process, and microstructure. The precipitation of fluorapatite crystalline phase is universally recognised as the core structural marker of bioactivity for such glass-ceramics, and its ability to induce bone-like apatite formation and achieve tissue bonding has been systematically verified by extensive in vitro and in vivo experimental studies.

Aluminium ions (Al^3+^) were initially incorporated into the glass because they play a key role in material hardening and solidification [7]. However, concerns have arisen regarding Al^3+^-induced neurotoxicity [8,9,10] (e.g., Alzheimer’s disease, Parkinson’s disease) and associated bone mineralisation defects. The results of clinical studies have demonstrated that the release of Al^3+^ from materials that have been implanted for an extended period may induce neurotoxicity. In order to mitigate the risks associated with aluminium (Al), researchers have sought to substitute Al with elements such as boron (B). As Zhang et al. [11] discovered, while B substitution has the capacity to reduce Al content, it concomitantly weakens the glass network and decreases the crystallisation temperature by up to 105 °C (at x = 25 mol%), a phenomenon that is disadvantageous for high-temperature sintering processes. Consequently, there is an urgent need to develop novel substitute elements that simultaneously ensure biosafety and thermal stability. As reported by Boyd et al. [12], zinc-based glasses have been shown to exhibit antibacterial activity against Streptococcus mutans and Actinomyces viscosus. Mokhtari S et al. [13] evaluated the antibacterial properties of copper (Cu^2+^)-doped SiO_2_–ZnO–CaO–SrO–P_2_O_5_ glass systems, demonstrating that Cu^2+^ exerts antibacterial effects against microorganisms such as Escherichia coli and Staphylococcus aureus. A plethora of studies have previously investigated the therapeutic effects of various non-traditional ions, including zinc (Zn^2+^) [14], strontium (Sr^2+^) [15], zirconium (Zr^4+^) [16], gallium (Ga^3+^) [17], and titanium (Ti^4+^) [18].

Germanium (Ge) has been identified as a promising alternative with distinct advantages. Germanium dioxide (GeO_2_), a glass former analogous to silica (SiO_2_), has been shown to enhance the chemical resistance of glasses and to substitute for aluminium (Al), thereby reducing the Al content in calcium alumino-silicate glasses. Firstly, organic Ge compounds have been used clinically as immunomodulators. Ge compounds have been shown to inhibit cancer progression, and inorganic Ge compounds can enhance cellular radiosensitivity [19]. Secondly, Ge^4+^ typically functions as a network former in glasses, existing as [GeO_4_] structural units that can theoretically isomorphically substitute Si^4+^ within the glass structure. GeO_2_ has been observed to form high-strength [GeO_4_] tetrahedra, which are theorised to enhance network polymerisation. Substitution of Al for Ge has been demonstrated to alter the content of bridging oxygens (BOs) and non-bridging oxygens (NBOs) in the glass structure, thereby regulating glass solubility [20,21]. Despite the extensive utilisation of Ge in the field of optical glasses [22,23], the precise mechanism of action of Ge in bioactive fluoro-alumino-silicate systems remains to be elucidated. Furthermore, the impact of Ge substitution for Al on the calcium–silicon–phosphorus network has been scarcely documented.

The present study focuses on the effect of Ge substitution for Al on the formation and properties of calcium fluoro-alumino-silicate glasses and glass-ceramics. The substitution was executed at varying molar percentages, with percentages of 0%, 20%, 40%, and 60% relative to the aluminium content. The characterisation of the samples was conducted through the utilisation of DSC, SEM, and EDS, complemented by XRD analysis. The objectives are twofold: firstly, to reveal the coordination state transition of Ge and its regulatory effect on phase composition, and secondly, to provide theoretical support for the design of novel dental bioceramics with low toxicity and high mechanical strength.

## 2. Materials and Methods

### 2.1. Raw Materials and Preparation

A calcium fluoro-alumino-silicate glass with the composition 4.5SiO_2_-3Al_2_O_3_-1.5P_2_O_5_-3CaO-2CaF_2_ was used as the base material. The molar compositions of the Ge-substituted glasses are listed in Table 1. Powders of SiO_2_, Al_2_O_3_, P_2_O_5_, natural CaF_2_, GeO_2_, and CaCO_3_ were manually mixed by shaking for 30 min and then transferred to a platinum–rhodium (Pt-5% Rh) crucible. The crucible was placed in an electric furnace and heated at 1450 °C for 1.5 h. The glass melt was then water-quenched to prevent phase separation and crystallisation. Finally, all glasses were ground into fine particles (<45 μm) using a gyro mill [24].

Four glass compositions were selected for crystallisation studies. Two grams of glass powder were compressed into a mould, thus creating test pellets for the subsequent heat treatment. The pellets were subsequently placed in a platinum crucible and heated in a muffle furnace. The samples were initially heated from room temperature to 700 °C at a constant heating rate of 10 °C/min and held at 700 °C for 1 h for isothermal nucleation. Thereafter, the samples were further heated to 1100 °C at the same heating rate of 10 °C/min and held at 1100 °C for 1 h to complete crystallisation. After the heat treatment, all samples were cooled to room temperature via furnace cooling, with an average cooling rate of approximately 3–5 °C/min. Melting at 1450 °C for 1.5 h ensures complete melting and uniform mixing of high-melting oxides, while suppressing excessive F and P volatilisation [25]. A unified two-step thermal treatment (700 °C nucleation for 1 h, 1100 °C crystallisation for 1 h) was used for all samples to rule out thermal history interference and enable direct comparison of Ge substitution effects. The 700 °C nucleation temperature surpasses the maximum Tg of undoped glass to guarantee adequate nucleation for all groups; 1100 °C lies above all samples’ secondary crystallisation peaks to fully precipitate FAp, anorthite and AlPO_4_. Even though Ge doping lowers Tg and crystallisation peaks, sample-specific heat schedules were not adopted to avoid extra confounding thermal variables.

### 2.2. Characterisation Methods

The thermal transitions of the glasses were analysed using a Netzsch 404C DSC with matched Pt-Rh crucibles (Germany). Approximately 20 milligrams of each sample powder was heated to 1200 °C at a rate of 10 °C/min and subsequently air-cooled. The glass transition temperature (Tg) and peak crystallisation temperature (Tp) were determined. As previously documented, 4.5SiO_2_-3Al_2_O_3_-1.5P_2_O_5_-3CaO-2CaF_2_ glass demonstrates notable surface nucleation; consequently, fine powder particles with a diameter of less than 45 μm were utilised in this study. Glass-ceramics were characterised by XRD using an X’Pert PRO MPD diffractometer (The Netherlands) with a Cu target (λ = 1.5406 Å), operating at 40 kV and 40 mA, with a scanning range of 5~90° and a step size of 0.02°.

Prior to the analysis by scanning electron microscopy (SEM), the glass-ceramic blocks were divided into smaller pieces using a diamond blade cutter and then mounted using epoxy resin. Samples were ground and polished using a polishing machine (DAP-7 and Pedemin-S, Denmark), with a final polishing step using a liquid diamond suspension (particle size < 1 μm). Following the polishing stage, all glass-ceramics were etched with 10% hydrofluoric acid (HF) and sputter-coated with gold using an SB250 coater. Morphological observation and elemental composition analysis were performed using a Hitachi SU8010 (Japan) field-emission scanning electron microscope equipped with an energy-dispersive X-ray spectroscopy (EDS) detector. All tests were carried out at an accelerating voltage of 20 kV and a working distance of 8.0 mm. For EDS point quantitative analysis, each measurement point was acquired with a live counting time of 60 s to ensure sufficient counting statistics and analytical accuracy.

## 3. Results and Discussions

### 3.1. XRD Analysis

**Figure 1:**
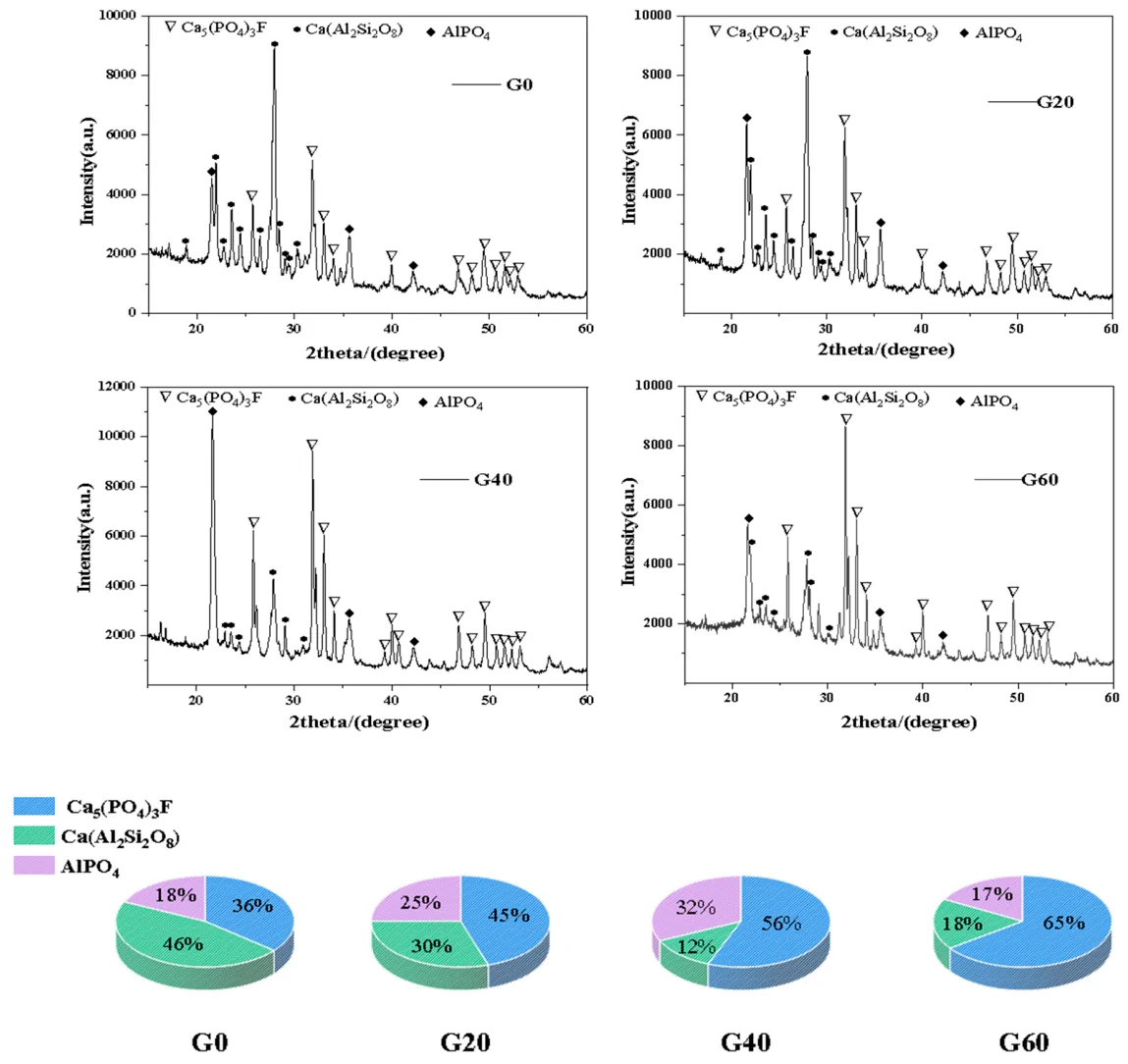
XRD results of G0, G20, G40, and G60 germanium-substituted glasses, 89-1460 (Ca(AI_2_Si_2_0_8_), 75-1072 (AlPO_4_), 71-0880 (Ca_5_(PO_4_)_3_F).

As illustrated in Figure 1, the XRD patterns and the corresponding quantitative phase composition pie charts of quenched glasses with varying Ge substitution levels (0 mol%, 20 mol%, 40 mol%, and 60 mol%) are presented. Rietveld refinement was utilised for the semi-quantitative analysis of XRD data. This method involves the calculation of the mass fraction of different crystalline phases by fitting the peak shape, position, and intensity, in combination with the reference intensity ratio (RIR) of each phase. For G0 (0 mol% Ge), sharp diffraction peaks are observed, with FAp accounting for 36%, anorthite for 46%, and AlPO_4_ for 18%. In G20 (20 mol% Ge substitution), the proportion of FAp increases to 45%, anorthite decreases to 30%, and AlPO_4_ increases to 25%. The diffraction peak intensity of FAp is significantly enhanced in comparison with G0, while that of anorthite is slightly weakened and AlPO_4_ is moderately strengthened without peak shift. It is evident that as the Ge substitution increases to 40 mol% (G40), there is a substantial change in the XRD pattern. It is evident that the FAp content has increased to 56%, while the proportion of anorthite has decreased to 12%, and AlPO_4_ has decreased to 22%. The FAp diffraction peak continues to strengthen, the anorthite peak is greatly weakened, and the AlPO_4_ peak begins to decline. With Ge substitution further increased to 60 mol% (G60), anorthite only accounts for 18%, AlPO_4_ for 17%, and FAp reaches 65%. The diffraction signal of AlPO_4_ is only observed at a low intensity, while the FAp diffraction peak is the strongest.

### 3.2. DSC Analysis

**Figure 2:**
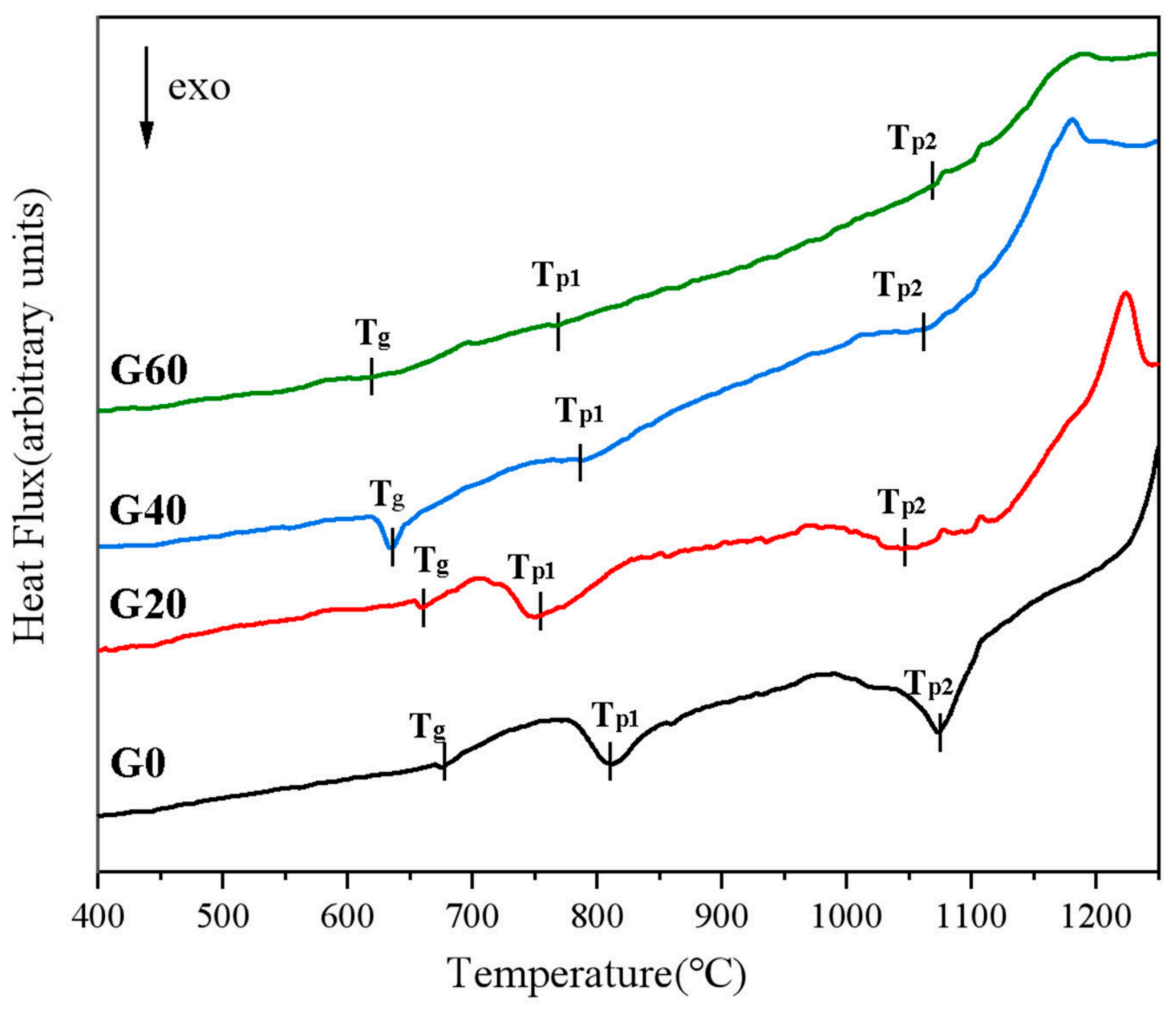
DSC traces of glasses with different Ge substitution levels; samples were heated at a heating rate of 10 °C/min.

As demonstrated in Figure 2, the glass samples with varying Ge substitution levels (G0 to G60) manifest significant regular changes in thermal behaviour, primarily characterised by a decrease in Tg with increasing Ge substitution. G0 has the highest Tg of 677.8 °C, with a sharp and intense peak crystallisation temperature (Tp1) at ~810.7 °C corresponding to FAp, and a second crystallisation peak (Tp2) at ~1075.1 °C corresponding to anorthite. In the absence of Ge substitution, Al^3+^ stabilises the formation of [AlO_4_] tetrahedra, thereby engendering a favourable network environment conducive to the precipitation of crystalline phases such as FAp and anorthite.

The glass transition temperature and crystallization temperature of germanium substituted glass are shown in Table 2. As Ge substitution reaches 20 mol% (G20), a decrease in Tg from 677 °C to 660 °C is observed, accompanied by a substantial decline in Tp1 of FAp from 810.7 °C to 749.4 °C. Notwithstanding the temperature reduction, the crystallisation peak persists as a distinct entity. Incorporation of low-content Ge^4+^ into the Al-O-Si network occurs as tetracoordinate [GeO_4_] tetrahedra. Ge^4+^ does not require additional Ca^2+^ for charge balance, and this only results in a slight weakening of the network bond energy. This not only reduces the thermal transition threshold of the glass, facilitating energy-saving sintering processes but also preserves the network’s ability to support the precipitation of crystalline phases, with no significant interference to the crystallisation kinetics. As the Ge substitution increases further to 40 mol% (G40), the glass transition temperature (Tg) decreases to 635.8 °C, and the crystallisation peak becomes significantly broadened with drastically reduced intensity. It has been demonstrated that an excess of Ge^4+^ has the capacity to disrupt the continuity of the Al-O-Si network. It is evident from the DSC curve that, upon reaching Ge substitution of 60 mol% (G60), there is an absence of any discernible peak corresponding to crystallisation. The aggregation of a large number of [GeO_4_] tetrahedra leads to excessive relaxation of the glass network, and the growth substrate for the Ca-P-F-rich droplet phase crystallisation is lost.

### 3.3. Scanning Electron Microscopy and Energy–Dispersive Spectroscopy Analysis

**Figure 3:**
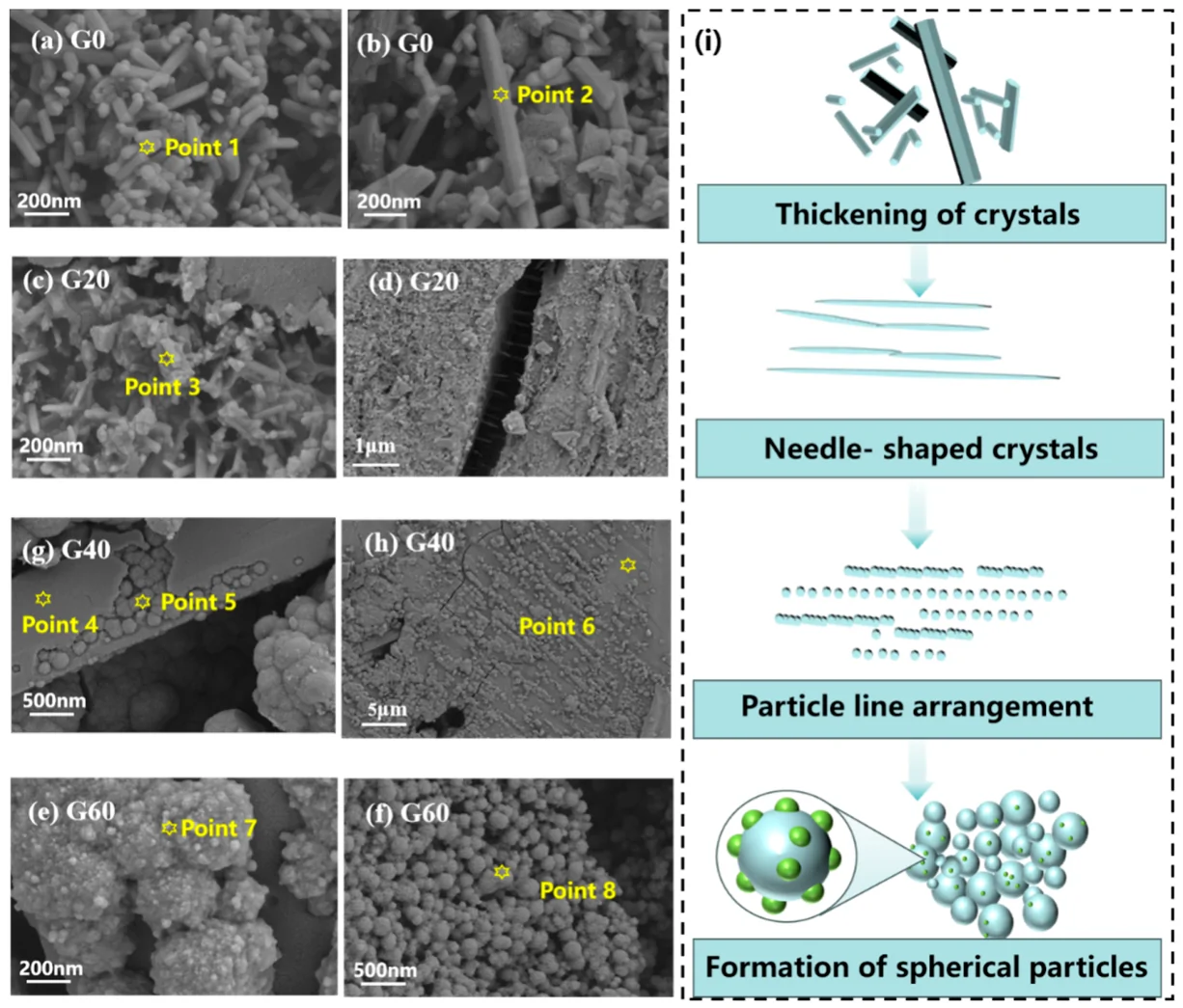
SEM morphologies of glass-ceramics, (**a**–**h**). (**i**) Illustration of the change of fluorapatite crystals.

It is shown in Figure 3 that the SEM micrographs and corresponding EDS analysis results of all Ge-substituted glasses after heat treatment at 1100 °C for 1 h. The regulatory effect of different Ge substitution levels on the microstructure of glass-ceramics exhibits significant regularity: the morphology of FAp gradually transforms from acicular/rod-like to spherical with increasing Ge substitution, and the crystallisation integrity decreases.

For G0 (0 mol% Ge), scanning electron microscopy (SEM) images (Figure 3a,b) demonstrate that a substantial quantity of regular acicular/rod-like crystals are distributed uniformly within the glass-ceramic matrix, exhibiting dimensions of approximately 5 μm in length, 0.5 μm in diameter, and an aspect ratio of 10:1. No obvious agglomeration is observed, and the crystals exhibit a certain degree of orientation. When combined with EDS analysis of Point 1 in Table 3, the atomic fractions of Ca, P, and F in the acicular/rod-like crystals are found to be 12.30%, 7.45%, and 12.56%, respectively, with a Ca/P ratio of ~1.65, which is consistent with the theoretical Ca/P ratio of FAp (1.67). This confirms that the acicular/rod-like crystals are pure FAp phase. Meanwhile, elevated concentrations of Al (8.50%) and Si (8.40%) have been detected in the matrix region, corresponding to the precipitation of anorthite and AlPO_4_ in XRD, thereby further verifying the completeness of the crystalline phase composition of G0. When Ge substitution increases to 20 mol% (G20), however, SEM images (Figure 3c,d) still show acicular/rod-like crystals as the main phase, with a size close to that of G0 (length ~4.5 μm, diameter ~0.45 μm). It has been demonstrated that only a limited number of fine particles manifest in localised regions, and the overall orientation remains unperturbed. EDS results (Point 4) demonstrate that the atomic fraction of F in the acicular crystal region is 55.24%, Ca is 12.71%, and the Ca/P ratio is 1.63, which is consistent with the compositional characteristics of FAp. Concurrently, 0.39% Ge was detected in the matrix region, indicating that Ge^4+^ has been uniformly dispersed in the glass network. However, the presence of low-content Ge does not compromise the stability of the Ca-P-F-rich droplet phase, and FAp can still facilitate oriented growth, thereby preventing any substantial morphological transformation. As Ge substitution reaches 40 mol% (G40), significant morphological transformation is observed in the SEM images (Figure 3e,f). This is characterised by the almost complete disappearance of acicular/rod-like crystals, which are replaced by irregular spherical particles in the matrix, with a diameter of approximately 0.8 μm.

The distribution uniformity is observed to decrease, and slight agglomeration occurs in localised areas. EDS analysis (Point 5) shows that the atomic fractions of Ca, P, and F in the spherical particles are 16.37%, 3.54%, and 52.10%, respectively, with a Ca/P ratio increased to 3.18. This deviates significantly from the theoretical Ca/P ratio of pure FAp (1.67), and the Si and Ge contents in this region are significantly higher than those in acicular FAp crystals. It indicates that the spherical particles are dominated by Si-Ge-rich glass matrix, with only trace amounts of incompletely crystallised FAp nanocrystal nuclei embedded inside, rather than independent incomplete FAp crystalline phases. Concurrently, the Al content diminished to 5.87%, while the Si content augmented to 2.21%. This outcome validates the hypothesis that elevated Ge content has engendered an adverse effect on the growth environment of specific crystalline phases, culminating in the exclusive presence of an incomplete FAp phase. As the Ge substitution increases to 60 mol% (G60), the SEM images (Figure 3g,h) reveal the presence of uniformly distributed spherical particles with a diameter of approximately 1 μm. There is an absence of acicular/rod-like crystals, and the particle surfaces are smooth without obvious grain boundary characteristics [26]. EDS analysis (Point 8) shows that the atomic fractions of Ca, P, and F in the spherical particles are 14.07%, 4.07%, and 51.00%, respectively, with the Ca/P ratio further increased to 3.46. The F content is found to be significantly higher than that of G0, which is related to the migration of residual F in the matrix to the particle surface after slight volatilisation of F during high-temperature melting. Concurrently, no discernible Al or P enrichment regions are identified, and the spherical particles manifest as an aggregated form of the amorphous glass matrix. It has been demonstrated that elevated levels of Ge content are capable of entirely inhibiting the oriented crystallisation of FAp.

### 3.4. Network-Forming Role of Germanium and Structure Regulation Mechanism

Based on the combined results of XRD, DSC and SEM-EDS, together with classical glass structure theory and previous reports, it can be reasonably inferred that Ge^4+^ mainly exists in the form of four-coordinated [GeO_4_] tetrahedral units in the amorphous glass network. This inference is supported by multiple examples of indirect evidence and is consistent with the first-principles calculation results of GeO_4_ glass structure reported by Chiroma et al. [27]. XRD patterns show no diffraction peaks assigned to Ge-bearing crystalline phases in all as-quenched glasses, confirming that Ge is homogeneously dissolved in the glass matrix rather than precipitated as independent crystalline phases. As GeO_4_ is a typical network-forming oxide, this result supports that Ge incorporates into the silicate network as tetrahedral structural units. The monotonic and moderate decrease in Tg with increasing Ge substitution revealed by DSC also matches the structural feature of tetrahedral Ge incorporation. If Ge acted as a network modifier in six-coordinated form, it would induce more severe network depolymerisation and a much sharper drop in Tg. The observed gradual decline in Tg corresponds to the isomorphous substitution of [AlO_4_] by [GeO_4_] tetrahedra, which only moderately reduces the average bond energy of the glass network. The regulation of the glass network structure exhibits a differentiated pattern: low substitution levels optimise thermal processability, while high substitution levels damage the crystallisation support capacity. This fundamental disparity is rooted in the intrinsic variations in charge balance and network integration between Ge^4+^ and Al^3+^. The disparities in atomic weight and ionic charge between Ge and Al have the potential to influence the bond energy of the glass network. Al^3+^ requires charge balance by Ca^2+^: each [AlO_4_] tetrahedron requires one Ca^2+^ to form a continuous Al-O-Si network, resulting in a high degree of polymerisation and stability of the glass structure [28]. In the event of Ge^4+^ substituting for Al^3+^, Ge^4+^ is able to directly incorporate into the network as tetracoordinate [GeO_4_] without additional charge balance. Ge^4+^ integrates uniformly into the glass network structure as [GeO_4_] tetrahedra, connecting with [SiO_4_] and [AlO_4_] tetrahedra through bridging oxygen bonds to form a continuous glassy network, without phase separation or crystallisation. Consequently, no diffraction peaks corresponding to Ge-based crystalline phases are observed in the XRD patterns. This network integration mode is a typical behaviour of Ge as a glass former, similar to the role of Si^4+^ in silicate glasses. The subject of this study is a substance that strengthens network polymerisation by constructing [GeO_4_] tetrahedra, while regulating network bond energy and thermal stability. The substance exists as a glassy phase at grain boundaries or in the matrix. Al in glasses frequently exists in a tetracoordination or hexacoordination configuration, while Ge has been observed to exhibit a preference for tetracoordination. Substitution effects a modification of the proportion of bridging oxygen bonds in the network, thereby exerting a direct effect on both the glass transition temperature and the crystallisation activation energy.

In the glass system with Ge substitution levels of 0–20 mol%, the coordination number transition of Ge^4+^ from [SiO_4_] to [GeO_4_] is the driving force behind amorphous phase separation, and Ge^4+^ incorporates moderately into the Al-O-Si network through “site-filling”. At this stage, the content of Ge^4+^ is minimal, resulting in only a slight dilution of the Al-O bonds without compromising network continuity. The reduction in glass transition temperature (Tg) from 677.8 °C to 616.8 °C is attributable to the weakening of part of the network bond energy (lowering the sintering threshold and optimising processability). Concurrently, the network’s constraint capacity on the Ca-P-F-rich droplet phase is retained. These droplet phases then prioritise ensuring the stable precipitation of crystalline phases such as FAp and anorthite [25], confirming that the network achieves a balance between easy sintering and functionality at this stage. It has been established that when the Ge substitution exceeds 40 mol%, there is a significant loss of integrity of the Al-O-Si network due to the presence of excessive Ge^4+^. The frequency of occurrence (FO) of the first apatite (FAp) increases from 45% to 65%, whilst the proportion of anorthite (An) decreases from 30% to 18%, and the proportion of AlPO_4_ falls back from 25% to 17%. This stage has been shown to enhance the process of FAp crystallisation, whilst concomitantly impeding the formation of anorthite and AlPO_4_ at elevated substitution levels. Conversely, the decline in Al content prompts Si^4+^ to predominantly bind with Ge^4+^, thereby yielding a Si-Ge-rich matrix phase. Concurrently, the initial Al-O-Si cross-linked structure undergoes dissolution. Quantitative EDS analysis of the glass indicates a significant increase in the Ca/P ratio from 1.6 to 3.5.

In instances where Ge substitution exceeds 40 mol% (40–60 mol%), there is a progressive destruction of the Al-O-Si network, leading to a decline in Al content to 1.2 mol% (G60). This is inadequate to facilitate the crystallisation of anorthite, resulting in a residual content of only 18%. AlPO_4_ accounts for a mere 17% due to the substantial deficiency of Al^3+^. Conversely, FAp, facilitated by the uninterrupted provision of Ca^2+^ and the regulation of the network by Ge^4+^, exhibits a notable increase to 65%, thereby ensuring the sustained strengthening of crystallisation. At this stage, although the glass network exhibits a certain degree of relaxation due to the high Ge content, the strong crystallisation ability of FAp compensates for the slight weakening of the network structure, enabling the material to maintain good structural stability [29].

From the perspective of material properties, low content only moderately regulates the network, while high content excessively damages the structure. The regulatory effect of low Ge substitution (G20) is the most practically valuable. A 20% reduction in Al content has been shown to have a beneficial effect on reducing toxicity risk. In addition, this reduction has been found to result in a moderate weakening of the network to lower Tg for easier sintering, while retaining the structural support required for crystalline phases. Conversely, elevated Ge substitution (≥40 mol%) has been observed to result in a further reduction in Tg, accompanied by excessive network relaxation, leading to the dissolution of functional crystalline phases. The material is subject to a loss of bioactivity and mechanical reinforcement phases, thus failing to meet medical requirements. This mechanism is consistent with the SEM observation that G20 retains acicular/rod-like FAp, while G40 and G60 transform into spherical amorphous particles. Collectively, these findings confirm that the regulation of the glass network by Ge must be controlled within a low substitution range.

### 3.5. Germanium-Induced Phase Separation and Fluorapatite Morphology Evolution

The combined SEM-EDS, XRD, and DSC results indicate that Ge substitution regulates the phase separation behaviour and the morphology of fluorapatite (FAp). The spherical particles under scrutiny are not independent FAp crystalline phases, but composite aggregates dominated by Si-Ge-rich glassy matrix, with trace amounts of incompletely crystallised FAp nanonuclei and residual auxiliary phases (AlPO_4_) embedded inside. This is consistent with the EDS result that the Ca/P ratio of spherical particles greatly deviates from the stoichiometric ratio of pure FAp: the detected Ca and P elements mainly come from the dispersed trace FAp residual nuclei, while the matrix part is composed of silicate glass network, resulting in an overall elevated Ca/P ratio. Noteworthily, these spherical particles are also essentially different from the Ca-P-F-rich droplet phase in as-quenched glasses. The droplet phase is a metastable phase-separation structure with concentrated Ca-P-F components at the nanoscale, which can induce FAp oriented crystallisation [30]. In contrast, the micron-sized spherical particles are glass matrix aggregates formed after the droplet phase collapses under high Ge content, which have lost the ability to support oriented crystal growth. EDS analysis indicates that the contents of Ge and Si in spherical particles are considerably higher than those in the acicular/rod-like FAp region. Furthermore, these spherical particles do not exhibit the typical Ca/P ratio of FAp (1.63–1.65), thereby confirming that their core component is the glassy phase rather than crystalline FAp. When Ge substitution is ≥40 mol%, the presence of excessive [GeO_4_] tetrahedra disrupts the continuity of the Al-O-Si network, thereby replacing the original rigid Al-O-Si cross-linked structure with a more flexible Si-Ge-rich network. The relaxation degree of the glass network is significantly increased, the number of non-bridging oxygen bonds increases, and the matrix fluidity is enhanced. The substitution of high Ge content has been shown to have a significant impact on the Al content, with Al_2_O_3_ being present in a mere 1.2 mol% of G60. This has been demonstrated to result in the complete collapse of the Al-Si-Ca-rich and Al-P-rich droplet phases, which have been identified as the primary support for the crystallisation of anorthite and AlPO_4_.

Auxiliary phases lose stable nucleation conditions and only present trace dispersed fine grains. During high-temperature sintering and cooling, the glass matrix spontaneously evolves into a low-surface-energy thermodynamic morphology. Moderate Ge substitution enhances glass fluidity via network relaxation, eliminating the rigid crystalline framework requirement during cooling [31]. Trace residual fine grains act as nucleation cores, driving the glass matrix to aggregate, contract, and form spherical particles under surface tension.

The Ge substitution content dominates glass phase separation and FAp growth behaviour. At low Ge substitution (0–20 mol%), Ge disrupts the homogeneity of the Al–O–Si network. Since Ge exhibits better compatibility with Si than Al, Si preferentially bonds with Ge to form the main matrix phase [32]. The reduced Al weakens its binding with Ca, enabling Ca and P to detach from the network and form uniformly dispersed nanoscale (50–100 nm) Ca–P–F-rich droplet phases. These droplets possess a Ca/P ratio of 1.63–1.65, close to the theoretical FAp value (1.67), providing a favourable nucleation environment for FAp. Benefiting from the lowest lattice energy of the FAp (001) plane, crystals preferentially grow longitudinally along the [001] direction, forming a stable acicular/rod-like morphology. At 20 mol% Ge substitution, Ge incorporates into the network as [GeO_4_] tetrahedra, slightly weakening Al–Ca binding but maintaining uniform droplet distribution and stable Ca/P ratio; thus, FAp retains its oriented acicular structure [33].

High Ge substitution (≥40 mol%) induces excessive phase separation and deteriorates FAp crystallisation. At 40 mol% Ge, intense phase separation destroys droplet phase stability, causing droplet agglomeration and collapse [34]. The excessive Ge further occupies Si sites and expels Al from the network, drastically reducing Al–Ca bonding and sharply increasing the droplet Ca/P ratio to 3.18. The imbalanced Ca/P stoichiometry terminates FAp oriented growth along (001), leading to random crystal growth and the formation of ~0.8 μm spherical particles with poor crystallinity [35]. The DSC results show only one broad crystallisation peak, indicating the disappearance of secondary crystalline phases (anorthite) and suppressed multi-step crystallisation caused by over-separation.

At 60 mol% Ge substitution, phase separation is completely suppressed, and Ca–P–F-rich droplet phases fully disappear. The microstructure consists solely of uniform ~1 μm spherical amorphous particles, with a further elevated Ca/P ratio (3.46) and surface-enriched F element. No localised Ca/P aggregation is observed, and FAp crystallisation is entirely inhibited.

Increasing Ge content reduces the initial crystallisation temperature and modulates FAp crystallisation kinetics. Low-Ge systems exhibit moderate nucleation density and matched activation energy, supporting stable FAp oriented growth and high crystallinity. In high-Ge systems, excessive phase separation produces isolated Ca-rich spherical domains, shortens Ca diffusion pathways, and increases crystallisation resistance, ultimately inducing morphological transformation and crystallinity loss [36]. From an application perspective, the 20 mol% Ge-substituted sample presents optimal bioactivity, with acicular FAp mimicking bone hydroxyapatite and improving bone tissue bonding. In contrast, high-Ge-derived spherical amorphous particles lose bioactivity and cannot meet dental and bone repair requirements [37].

## 4. Conclusions

In this study, calcium fluoro-alumino-silicate glasses with varying Ge substitution levels (0–60 mol%) were prepared via the melting–quenching method. A systematic investigation was conducted into the effects of Ge substitution for Al on the structure, thermal behaviour, crystallisation characteristics, and mechanical properties of glasses and glass-ceramics. This investigation utilised a range of analytical techniques, including DSC, XRD, and SEM-EDS, among others. The primary conclusions that can be drawn from this analysis are as follows:

As the Ge substitution level increases, there is a concomitant increase in FAp, which rises from 36% to 65%. Anorthite, conversely, decreases from 46% to 18%. AlPO_4_ displays a two-phase behaviour, first increasing and then decreasing, reaching a peak content of 25%. Ge^4+^ incorporates into the glass network as tetracoordinate [GeO_4_] tetrahedra without forming independent Ge-based phases. It has been demonstrated that this process does not necessitate the addition of further Ca^2+^ ions to ensure charge balance, and the Ca^2+^ ions that are released during this process provide sufficient raw materials for the subsequent crystallisation of FAp. Its high compatibility with Si^4+^ has been demonstrated to have an indirect inhibitory effect on the formation of anorthite. Substitution of the elements in question has been demonstrated to reduce the glass transition temperature (Tg) from 678 °C to 617 °C, with a concomitant decrease in the peak crystallisation temperature. Ge^4+^ has been demonstrated to be capable of reconstructing the Al-O-Si network. At low substitution levels, it has been shown to slightly weaken the network bond energy. Conversely, at high substitution levels, it has been observed to disrupt the network continuity, forming a flexible Si-Ge-rich network. This has been shown to optimise sintering processability and ensure the continuous crystallisation of FAp.

At low Ge substitution levels (≤20 mol%), stable phase separation supports the regular crystallisation of FAp, anorthite, and other phases, with no spherical particles formed. At high Ge substitution levels (≥40 mol%), however, the Al-Si-Ca-rich droplet phase corresponding to anorthite collapses due to insufficient Al content, the Al-P-rich droplet phase shrinks, and only the Ca-P-F-rich droplet phase stably exists. The Si-Ge-rich glassy matrix has been observed to aggregate around residual trace auxiliary phases due to network relaxation and enhanced fluidity, forming spherical particles driven by the minimum thermodynamic surface energy. The optimal Ge substitution level is determined to be 20 mol%, which has been shown to reduce Al^3+^ content by 20% to mitigate toxicity risk, moderately lower Tg for enhanced sintering, and retain the synergistic effect of FAp, anorthite, and AlPO_4_, thereby integrating low toxicity, sinterability, high bioactivity, and good mechanical properties. It has been established that high Ge substitution (i.e., ≥40 mol%) is likely to result in a decline in mechanical properties, thereby hindering the ability to comply with medical requirements.

This study addresses the existing lacunae in the mechanism of Ge substitution for Al in calcium fluoro-alumino-silicate bioactive glass systems, elucidates the fundamental advantages of low Ge content substitution (20 mol%), and provides both data support and theoretical basis for the development of low-Al, easy-to-sinter, high-bioactivity bioactive glass-ceramics for dental and bone repair. Subsequent research should concentrate on in vitro analyses of cellular toxicity, in vivo bone integration, and mechanical property testing of the G20 group, with a view to providing further evidence for its clinical application potential.

## Figures and Tables

**Table 1:** Molar composition of germanium substituted aluminosilicate glass.

	SiO_2_	Al_2_O_3_	P_2_O_5_	CaO	CaF_2_	GeO_2_
G0	4.5	3	1.5	3	2	0
G20	4.5	2.4	1.5	3	2	0.6
G40	4.5	1.8	1.5	3	2	1.2
G60	4.5	1.2	1.5	3	2	1.8

**Table 2:** Glass transition temperature and crystallisation temperature of germanium-substituted glasses.

	Tg	Tp1	Tp2
G0	677.8	810.7	1075.1
G20	660.6	749.4	1046.6
G40	635.8	787.6	1060.2
G60	616.8	770.1	1077.2

**Table 3:** Chemical composition (At.%) from EDS analyses.

At.%	Point 1	Point 2	Point 3	Point 4	Point 5	Point 6	Point 7	Point 8
O	50.66	51.78	51.00	18.67	19.26	17.32	21.09	22.00
F	12.56	12.20	8.66	55.24	52.10	59.72	51.28	51.00
Al	8.50	8.12	8.75	5.92	5.87	0.21	8.22	7.02
Si	8.40	7.77	9.28	1.83	2.21	10.34	2.28	1.33
P	7.45	7.21	8.50	3.91	3.54	0.32	2.84	4.07
Ca	12.30	11.64	13.64	12.71	16.37	11.22	13.85	14.07
Ge	1.20	1.15	0.00	1.53	0.39	0.81	0.10	0.33

## Data Availability

The raw data supporting the conclusions of this article will be made available by the authors on request.

## References

[1] Freitas P., Tan J.L.T., Ishikawa K. (2025). Monolithic copper-doped carbonated apatite synthesized at low-temperature exhibits in vitro antibacterial effect and in vivo bone regenerative properties. Materialia.

[2] Ali R.H., Sghaier Z., Ageorges H., Salem E.B., Hidouri M. (2025). Sintering, mechanical and biological properties of Magnesium-Calcium Fluoro-Hydroxyapatites containing Zinc bioceramics for bone tissue engineering. Ceram. Int..

[3] Liu Q., Huang S.S., Matinlinna J.P., Chen Z.F., Pan H.B. (2013). Insight into biological apatite: Physiochemical properties and preparation approaches. BioMed Res. Int..

[4] Combes C., Cazalbou S., Rey C. (2016). Apatite biominerals. Minerals.

[5] Alkaron W., Almansoori A., Balázsi C., Balázsi K. (2024). A critical review of natural and synthetic polymer-based biological apatite composites for bone tissue engineering. J. Compos. Sci..

[6] Yotsova R., Peev S. (2024). Biological properties and medical applications of carbonate apatite: A systematic review. Pharmaceutics.

[7] Stamboulis A., Law R.V., Hill R.G. (2004). Characterisation of commercial ionomer glasses using magic angle nuclear magnetic resonance (MAS-NMR). Biomaterials.

[8] Bhattacharyya M.H., Jeffery E., Silbergeld E.K. (2023). Bone metabolism: Effects of essential and toxic trace metals. Toxicology of Metals.

[9] Abdalla S., Mishriky M., Abdel-Halim W., Abdalla M., Abdellah F. (2024). Occupational exposure to aluminum and cognitive performance. Egypt J. Occup. Med..

[10] McLachlan D.R., Fraser P.E., Jaikaran E., Lukiw W.J. (2023). Alzheimer’s disease and other aluminum-associated health conditions. Toxicology of Metals.

[11] Zhang S.Q., Stamboulis A., Ni W. (2019). The effect of boron substitution for aluminium on the microstructure of calcium fluoro-aluminosilicate glasses and glass-ceramics. J. Eur. Ceram. Soc..

[12] Boyd D., Li H., Tanner D.A., Towler M.R., Wall J.G. (2006). The antibacterial effects of zinc ion migration from zinc-based glass polyalkenoate cements. J. Mater. Sci. Mater. Med..

[13] Mokhtari S., Skelly K.D., Krull E.A., Coughlan A., Mellott N.P., Gong Y., Borges R., Wren A.W. (2017). Copper-containing glass polyalkenoate cements based on SiO_2_–ZnO–CaO–SrO–P_2_O_5_ glasses: Glass characterization, physical and antibacterial properties. J. Mater. Sci..

[14] Xie D., Feng D.S., Chung I.D., Eberhardt A.W. (2003). A hybrid zinc–calcium–silicate polyalkenoate bone cement. Biomaterials.

[15] Wren A., Boyd D., Towler M.R. (2008). The processing, mechanical properties and bioactivity of strontium based glass polyalkenoate cements. J. Mater. Sci. Mater. Med..

[16] Gu Y.W., Yap A.U.J., Cheang P., Khor K.A. (2005). Effects of incorporation of HA/ZrO_2_ into glass ionomer cement (GIC). Biomaterials.

[17] Keenan T.J., Placek L.M., Coughlan A., Bowers G.M., Hall M.M., Wren A.W. (2016). Structural characterization and anti-cancerous potential of gallium bioactive glass/hydrogel composites. Carbohydr. Polym..

[18] Dickey B.T., Kehoe S., Boyd D. (2013). Novel adaptations to zinc–silicate glass polyalkenoate cements: The unexpected influences of germanium based glasses on handling characteristics and mechanical properties. J. Mech. Behav. Biomed. Mater..

[19] Lin M.H., Hsu T.S., Yang P.M., Tsai M.Y., Perng T.P., Lin L.Y. (2009). Comparison of organic and inorganic germanium compounds in cellular radiosensitivity and preparation of germanium nanoparticles as a radiosensitizer. Int. J. Radiat. Biol..

[20] Serra J., González P., Liste S., Chiussi S., León B., Pérez-Amor M., Ylänen H.O., Hupa M. (2002). Influence of the non-bridging oxygen groups on the bioactivity of silicate glasses. J. Mater. Sci. Mater. Med..

[21] Yamanaka H., Nakahata K., Terai R. (1987). Structure of Na_2_O-TiO_2_-SiO_2_ glasses from the viewpoint of non-bridging oxygen measured by XPS. J. Non Cryst. Solids.

[22] Pan’kin D.V., Sukhanov V., Tver’yanovich Y.S., Churbanov F. (2017). Investigation of structure of GeS1.35 glasses with the use of isotopically enriched germanium and Raman scattering spectroscopy. J. Non Cryst. Solids.

[23] Çamiçi H.C., Guérineau T., LaRochelle S., Messaddeq Y. (2025). Optical investigation and thermal stabilization of germanium-gallium-containing bismuthate glasses towards NIR-MIR applications. Opt. Mater..

[24] Zhang S.Q., Stamboulis A. (2016). Effect of zinc substitution for calcium on the crystallisation of calcium fluoro-alumino-silicate glasses. J. Non Cryst. Solids.

[25] Denry I.L., Holloway J.A., Nakkula R.J., Walters J.D. (2005). Effect of niobium content on the microstructure and thermal properties of fluorapatite glass-ceramics. J. Biomed. Mater. Res. Part B Appl. Biomater..

[26] O’Flynn K.P., Stanton K.T. (2010). Nucleation and early stage crystallization of fluorapatite in apatite-mullite glass-ceramics. Cryst. Growth Des..

[27] Chiroma A. (2025). Understanding Germanium Coordination and Raman Signatures in GeO_2_ Glass Using First Principle Molecular Dynamics. Fane-Fane Int. Multidiscip. J..

[28] Thomsen R., Skibsted J., Yue Y.Z. (2018). The charge-balancing role of calcium and alkali ions in per-alkaline aluminosilicate glasses. J. Phys. Chem. B.

[29] Cui W.Y., Song X.L., Chen J.H., Chen Y., Li Y.Q., Zhao C.H. (2020). Adsorption behaviors of different water structures on the fluorapatite (001) surface: A DFT study. Front. Mater..

[30] Kamali M., Yekta B.E., Javadpour J., Heidari S. (2025). Effects of glass synthesis route on the characteristics of powder and properties of GICs in the calcium-fluoro-alumino-silicate system. Ceram. Int..

[31] Tao S., Ping Y., Huang F., Hua Y., Qiao P., Xu Z., Li Y., Ma H. (2023). Design of the YAG: Ce^3+^ phosphor in tellurite-germanate glass with high luminous efficiency for laser lighting. Ceram. Int..

[32] Zhang Y., Zhao J., Li D., Fan Z., Zhou S., Zhao F., Ye R., Hua Y., Huang L., Lei L. (2026). Defect-Engineered Multicolor Persistent Luminescent Germanate Glass Ceramic for Advanced Anti-Counterfeiting and x-Ray Imaging. Adv. Funct. Mater..

[33] Chen X., Mo D., Li M., Hu B., Hu Q., Ma R., Yuan B. (2026). Germanium-doping modification in photo-thermo-refractive glass. Ceram. Int..

[34] Lin X., Jiang X., Wang Z., Liu S., Liu L., Ning T., Jiang Y., Lu A. (2023). Crystallization behavior, thermal and fluorescence properties of germanate glass ceramic based on Ga_2_O_3_ replacing GeO_2_. Opt. Mater..

[35] Rasouli-Jouryabi S., Moosavi-Khoonsari E. (2026). Coupled Experimental Study and Thermodynamic Modeling of GeO_2_ and GeO_2_–CaO and GeO_2_–SiO_2_ Binary Systems. J. Am. Ceram. Soc..

[36] Rao P.T., Kumar N.R. (2026). PbO-induced modifications in the structural and optical properties of BaTiO_3_ ceramics. Appl. Phys. A.

[37] Darwish A.G., Farouk M.I., Abdelglil M.I., Abo-Mosallam H.A. (2026). Influence of Ti^4+^ on structure, mechanical, and dielectric performance of sustainable, lead-free CaO–MgO–P_2_O_5_–SiO_2_ glasses for energy storage. J. Mater. Sci..

